# Double Medial Elbow Bump: A Case Report of an Untreated Medial Epicondyle Fracture With Anterior Incarceration of the Fragment

**DOI:** 10.7759/cureus.66686

**Published:** 2024-08-12

**Authors:** Ajay Kamat, Ishwar S Dharmshaktu, Krishna Dev S Yadav, Ganesh S Dharmshaktu

**Affiliations:** 1 Department of Orthopaedics and Trauma, Sindhudurg Shikshan Prasarak Mandal (SSPM) Medical College and Lifetime Hospital, Sindhudurg, IND; 2 Department of Orthopaedics, Government Medical College Haldwani, Haldwani, IND; 3 Department of Orthopaedic Surgery, Government Medical College Haldwani, Haldwani, IND

**Keywords:** displaced fracture, neglected fracture, medial epicondyle fracture, traumatic injury, elbow

## Abstract

Medial epicondyle fractures are uncommon elbow injuries and require careful radiological evaluation for appropriate diagnosis and management. Missed or neglected medial epicondyle fractures, however, are reported as uncommon reports or small series. Incarceration of the medial epicondyle fragment within the elbow joint is often reported and poses therapeutic challenges. Severe displacement of the medial epicondyle fragment and its anterior incarceration within the soft tissues is a rare entity. Here, we report a two-month-old untreated case of an anteriorly displaced medial epicondyle fracture with anterior incarceration, presenting as a clinical bump adjacent to the native medial humeral condyle. This presentation of a double medial bump is uncommon and reported here for its rarity. The injury was finally managed with open reduction internal fixation of the displaced medial epicondyle fragment back to its native site along with the anterior transposition of the ulnar nerve. Good clinical outcome with full elbow range of motion and radiological union was achieved in the follow-up of 13 months.

## Introduction

Medial epicondyle fractures are uncommon pediatric elbow injuries, which constitute about 12-20% of all elbow injuries with the peak age range at 9-14 years [1,2]. Medial epicondyle fractures are associated with elbow dislocation in more than half of the cases with a few presenting with the incarceration of the fracture fragment into the elbow joint. Apart from this, other associated fractures or ulnar nerve injuries require identification and documentation [2]. Both acute or neglected presentations are reported and require appropriate management for optimal outcomes. Operative reduction and fixation are required for most displaced fractures to restore the elbow anatomy. Operative indications are also warranted for associated joint incarceration, ulnar nerve involvement, elbow instability, or high-demand athletes [1-3].

## Case presentation

**Figure 1:**
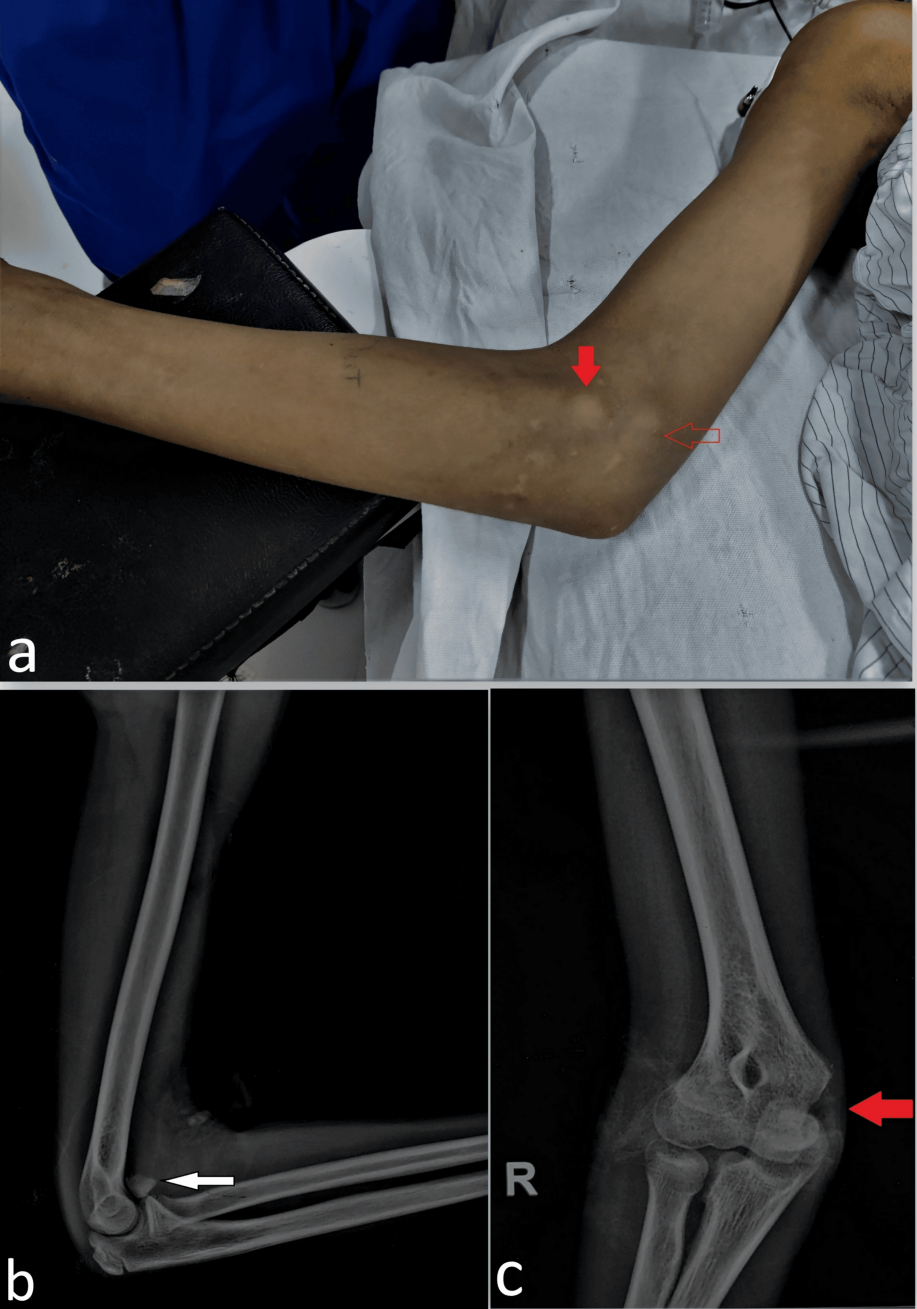
The clinical image (a) shows the presence of a double medial bump by the displaced medial epicondyle fragment placed anteriorly (solid red arrow) and the native humeral medial condyle bed situated posteriorly (hollow red arrow). The elbow radiograph at presentation shows a bony fragment anterior to the elbow joint (denoted by a white arrow) in the lateral view (b), and a defect at the normal location of the medial epicondyle region (c), suggesting an absence of the medial epicondyle from that area (denoted by a thicker red arrow).

A 13-year-old male adolescent presented to us with a palpable bump in the medial aspect of the elbow with a history of elbow injury two months back (Figure 1a). The elbow radiograph revealed a bony fragment anterior to the elbow joint along with a defect in the medial epicondyle region which suggested a displaced medial epicondyle fracture incarcerated anteriorly (Figures 1b, 1c). The same fragment was corresponding to the clinical anterior medial bump, while the native medial condyle bump was palpable posterior to it at its normal location. This overall medial double bump was a stark clinical feature. There was also a loss of terminal flexion and extension with an elbow arc of motion of 40-degree flexion to 20-degree short of full extension. However, no distal neurovascular deficit was noted. Old radiographs and injury paperwork were retrieved from the previous hospital.

**Figure 2:**
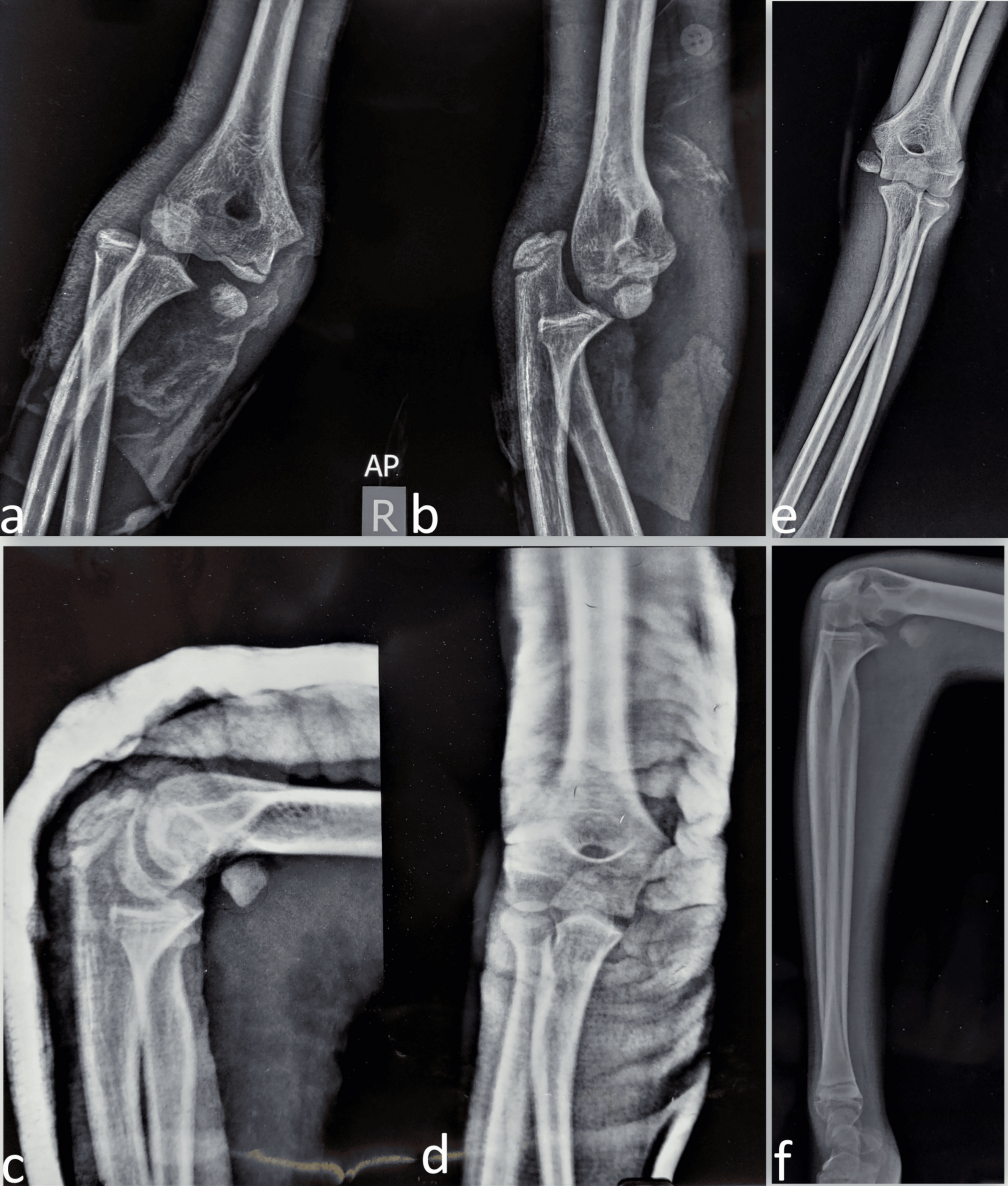
The previous radiographs show posterolateral elbow dislocation in the anteroposterior (a) and lateral (b) views. There is an associated medial epicondyle displaced fragment. The elbow dislocation was reduced well and radiographs show reduced elbow (c) given a plaster slab (d). The untreated medial epicondyle fracture is visible in the radiograph (e) after one month of the injury (f).

There was a history of a fall from a tree about two months back on the outstretched hand leading to pain, deformity, and inability to move the right elbow. He was rushed to a nearby hospital where he was diagnosed with posterolateral elbow dislocation following radiography (Figures 2a, 2b). No other injury or distal neurovascular deficit was present at that time. Closed reduction was attempted and was successful and he was given a plaster protection slab (Figures 2c, 2d). The presence of the associated fracture of the medial epicondyle was explained to the parents along with the need for operation, but due to personal issues, they refused it at that time and chose plaster treatment. His plaster was removed after four weeks and elbow active range of motion exercises were initiated. The elbow was stable at the time of presentation but the elbow movements were restricted as previously described. Based on the previous history of elbow injury, the diagnosis of displaced medial epicondyle fracture with an anterior incarceration of the fragment was made (Figures 2e, 2f).

**Figure 3:**
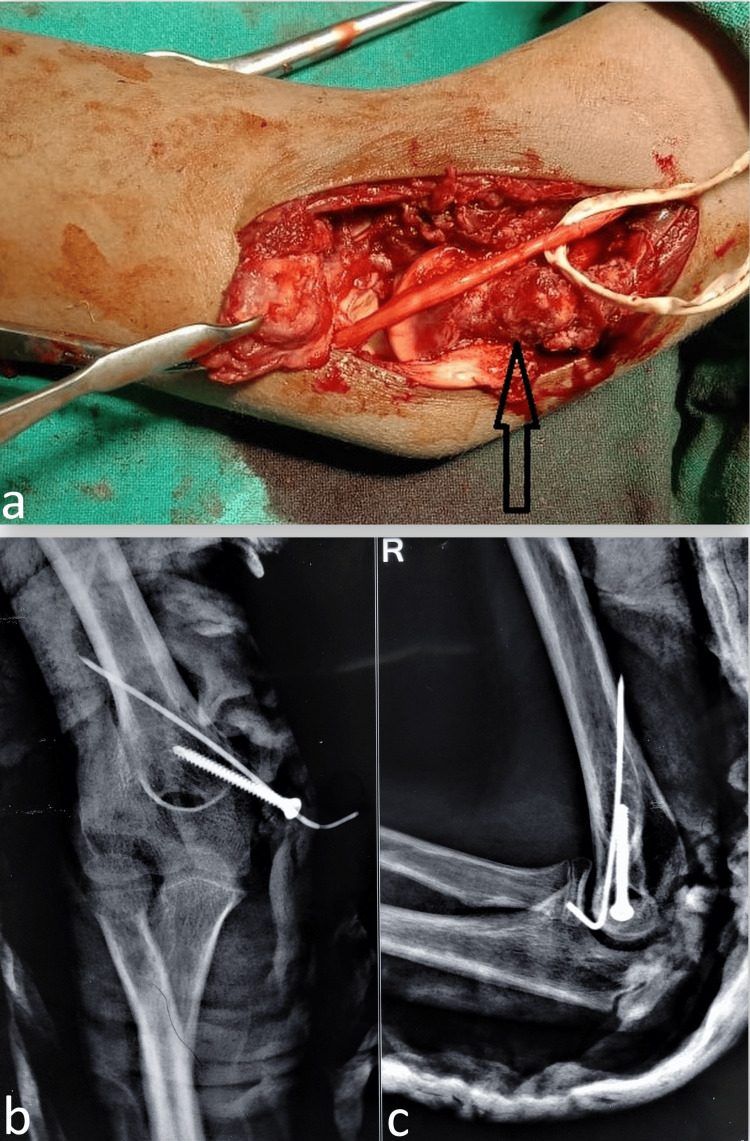
The intraoperative image shows the displaced medial epicondyle fragment (denoted by the tip of the bone lever) lying anteriorly (a) and exploration of the ulnar nerve done before the anterior transposition. The native bed (black arrow) was cleaned before the fixation of the medial epicondyle back to its normal location. The postoperative orthogonal radiograph shows the fixation of the fragment with a K-wire and a screw (b, c).

At our center, parental consent for open reduction and internal fixation was taken along with that of potential anterior transposition of the ulnar nerve if need be. A medial approach was used to access the fracture fragment that was found lying anteriorly within soft tissues and carefully dissected. The native attachment site was cleared of fibrous tissues and superficially nibbled to obtain a bleeding surface for better healing. The fragment with soft tissue and muscle attachments was stretched for a few minutes, provisionally placed at its normal site, and held with a K-wire (Figure 3a). A cannulated screw was introduced fixing the fragment with the bone adjacent to the previously placed K-wire and the same K-wire was retained as additional supportive fixation (Figures 3b, 3c).

The ulnar nerve was transposed anteriorly as per the standard method and the wound was closed in layers followed by a plaster protection slab. The perioperative period was uneventful, stitches were removed after 10 days, and plaster was continued for three weeks. After three weeks, the plaster slab and K-wire were removed in the outpatient clinic and the elbow range of motion was initiated. No immediate or remote complication of surgery was noted.

**Figure 4:**
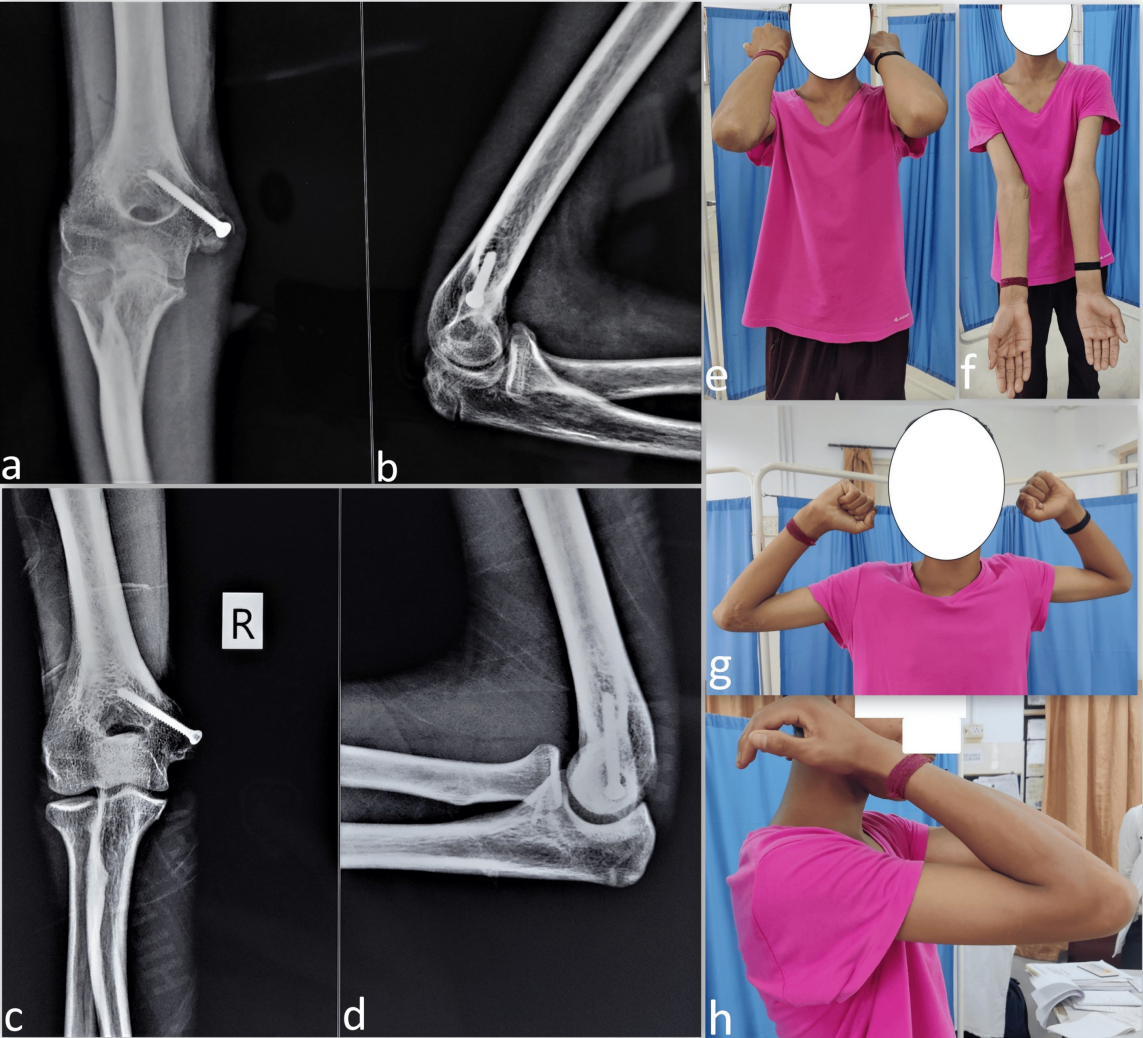
The radiograph at the 6th (a, b) and 13th-month (c, d) follow-up showing gradual union of the fracture along with good functional outcomes and regain of normal elbow movements with no elbow deformity (e, f) and full flexion-extension (g, h).

An uneventful and gradual fracture healing was noted in the follow-up at six months (Figures 4a, 4b). At the follow-up of 13 months, the fracture was completely united with no major complications (Figures 4c, 4d). The patient was performing all activities of daily living without deformity (Figures 4e, 4f) with a stable joint and painless full regain of elbow movements (Figures 4g, 4h).

## Discussion

Missed medial epicondyle fractures are uncommon and described sporadically in the literature. The medial epicondyle fractures are common in the setting of pediatric elbow dislocations and males are more commonly affected [1-4]. Our patient also was a male with a previously managed elbow dislocation but with an untreated displaced medial epicondyle fracture. The fractures may also be missed even after the reduction of the associated elbow dislocation and may present late with complications such as ulnar nerve symptoms [4,5]. Usually, ulnar nerve symptoms are late presentations and seldom seen in acute settings. Our case also had preoperatively intact ulnar nerve and ulnar nerve transposition was done as a protective step for potential future involvement. Missed or neglected injuries are rare but many believe that these injuries may be underreported [6].

Nonunion is high in conservatively managed cases at up to 90%, and for cases like ours with severe displacement, union without open reduction would not have been possible [7]. One-fifth of nonunion cases become symptomatic with pain, weakness, decreased range of motion, instability, or ulnar paraesthesia [8]. Our case had no pain but limited movement, and due to severe displacement, the risk of nonunion was high, thus warranting operative fixation. The fragment looked fixable but an optional excision of the fragment in case of non-fixable anatomy was also explained to the parents. Fixation is dependent on fragment size, if it is fixable with screws, then that is the better option. In cases with a small fragment, fragment excision followed by suture anchor fixation of the flexor pronator mass and medial ligamentous complex can also be utilized [9]. Despite the excision of the fragment utilized by many surgeons, open reduction and internal fixation is associated with good outcomes [10]. Our case also fared excellently with sound union and good clinical and functional outcomes.

Due to the common flexor origins and medial collateral ligament, the fracture displacement following a medial epicondyle fracture is usually distal and thus also within the joint. The superior and anterior displacement, as in our case, has not been reported to our knowledge. Hence, this report can enrich the literature with a rare injury pattern and its relevant details.

## Conclusions

Displaced medial epicondyle fractures are important injuries and care should be taken to not miss them with the help of careful radiological evaluation. Often in the setting of elbow dislocation, these fractures may result in intra-articular incarceration and require operative exploration and fixation. Untreated fractures with anterior incarceration are seldom described and may clinically present with a peculiar double medial bump. Displaced medial epicondyle fragments in these cases require open reduction and internal fixation along with the ulnar nerve transposition as a viable treatment method. Good functional outcomes can be expected with good union and early and compliant elbow physiotherapy.
